# Supplementary material for: Land use driven change in soil pH affects microbial carbon cycling processes
Source: Nat Commun. 2018 Sep 4;9:3591. doi: 10.1038/s41467-018-05980-1 (PMC6123395; doi:10.1038/s41467-018-05980-1)
Supplement: Supplementary file 1 — Supplementary Information [file 41467_2018_5980_MOESM1_ESM.pdf]

Supplementary Information

Land use driven change in soil pH affects microbial carbon cycling processes

Malik et al.

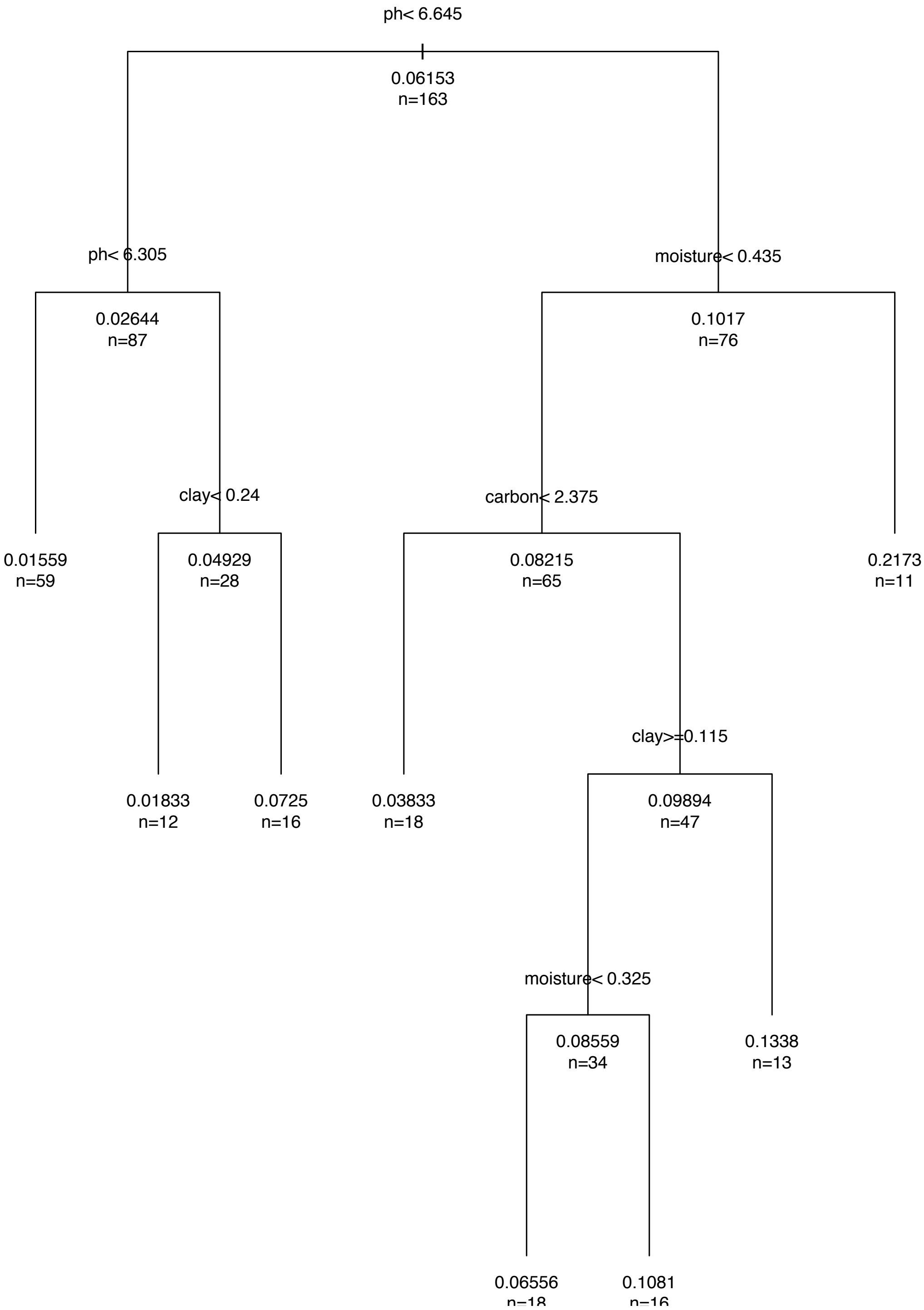

Supplementary Figure 1: Outcome of regression tree analysis. This was aimed at finding the best partitioning parameter explaining the variance in microbial CUE. The following edaphic factors were used in the test: soil moisture, pH, clay content, C and N concentration, and C: N ratio. Because CUE and edaphic factors interacted in a complicated non-linear way, assembling a single linear model was difficult. Therefore, we used this approach to partition the data into smaller sets that allows studying the interactions in a more manageable way.

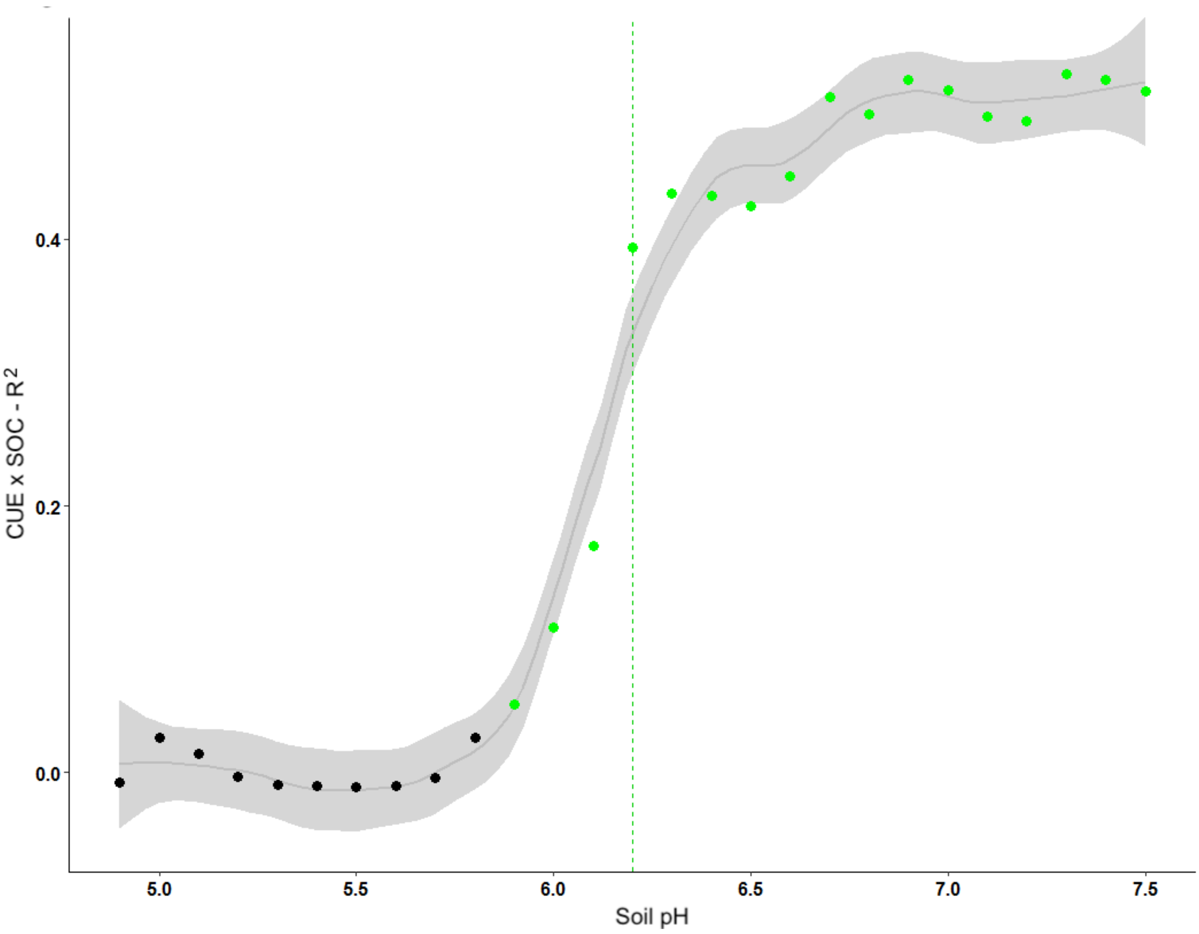

Supplementary Figure 2: Threshold determination by slope failure test. Distribution of the R^2 of the CUE-Soil C linear model across narrow intervals of soil pH (intervals of 1.4 units, increments of 0.1 units). Shaded area represents 95% confidence intervals around the trend line estimated using a t-based approximation (LOESS smoothing). Green points indicate significant linear regression ($p < 0.05$) whereas black points indicate a non-significant regression. At higher pH, better model fit was observed. But the relationship breaks down towards the lower range of soil pH, at a threshold where the slope fails. The threshold was estimated as the last pH unit (pH 6.2) before the R^2 drops dramatically.

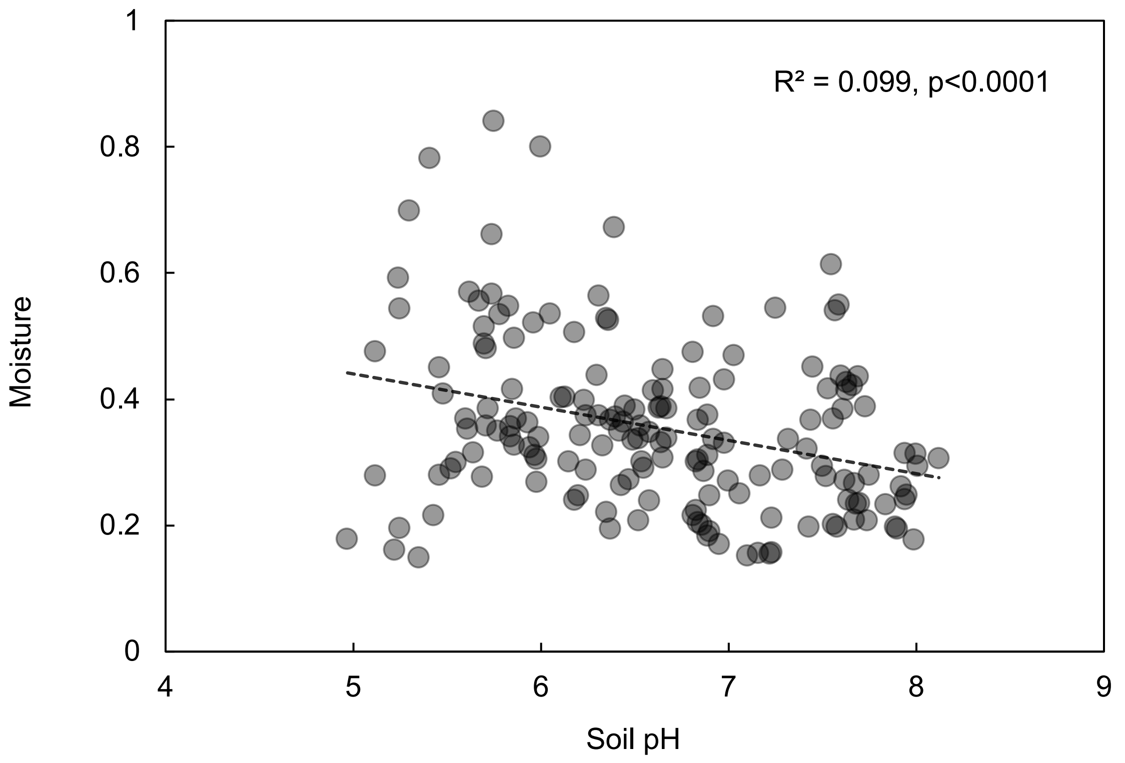

Supplementary Figure 3: Relationship between soil pH and moisture. Correlation of pH and moisture across all soils demonstrating that acidic soils are often also wet. Moisture was measured gravimetrically and presented here is the fraction of moisture relative to total weight.

Site Pair ID	Site location	Low intensity contrast			High intensity contrast			Reason for non-selection
		Management	Soil pH	Soil C %	Management	Soil pH	Soil C %	
22	Leicestershire	Unimproved grassland	6.8	5.6	Intensive: arable	6.1	2.9	Significant reduction in pH with intensification, contrary to broader trends
23	Devon	Intensive grassland	6.6	4.3	Intensive grassland	6.3	5.6	Recently established (2010) reseeding experiment contrasting different plant species on intensive grassland
24	Berkshire	Limed grassland with nutrients	7.9	3.1	Limed grassland, no nutrients	7.8	3.1	Experimental plots testing impact of nutrient amendments on grasslands, across different pH treatments (maintained through liming). Specific impacts on microbial traits are under further consideration separately.
25	Berkshire	Unlimed grassland with nutrients	5.2	2.7	Unlimed grassland, no nutrients	5.2	3.5	
26	Hertfordshire	Limed grassland with nutrients	7.2	3.7	Limed grassland, no nutrients	7.3	4.3	
27	Hertfordshire	Unlimed grassland with nutrients	5.5	2.8	Unlimed grassland, no nutrients	5.9	5.3	
28	Ceredigion	Unimproved grassland for at least 20 years	5.8	6.9	Intensive grassland	6.9	6.2	Insufficient field replication, n=2 for this contrast

Supplementary Table 1: Characteristics of excluded land use contrasts. Land use histories and edaphic properties of the 7 paired contrasts that were not included in the assessment of land use intensification effects.
